# Supplementary material for: Low-Frequency Magnetic Resonance Imaging Identifies Hand Joint Subclinical Inflammation in Systemic Sclerosis
Source: Diagnostics (Basel). 2022 Sep 6;12(9):2165. doi: 10.3390/diagnostics12092165 (PMC9497728; doi:10.3390/diagnostics12092165)
Supplement: Supplementary file 1 [file diagnostics-12-02165-s001.zip › diagnostics-1831013-supplementary.pdf]

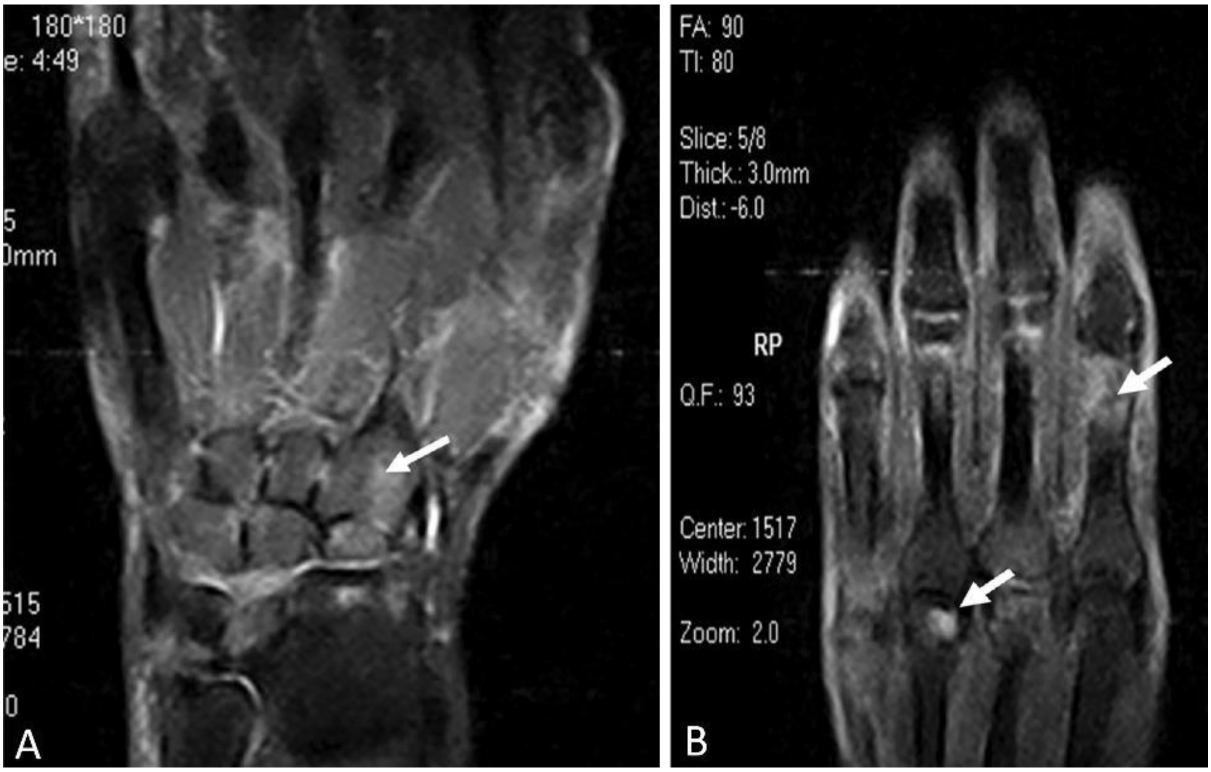

Supplementary Figure S1. (**A**) Bone edema in wrist presented as the zone of high signal values at T2w STIR cor SE sequence; (**B**) erosive lesions at left hand MCP IV and bone edema in PIP II with perivascular component at T2w STIR cor SE sequence in patient with SSc.

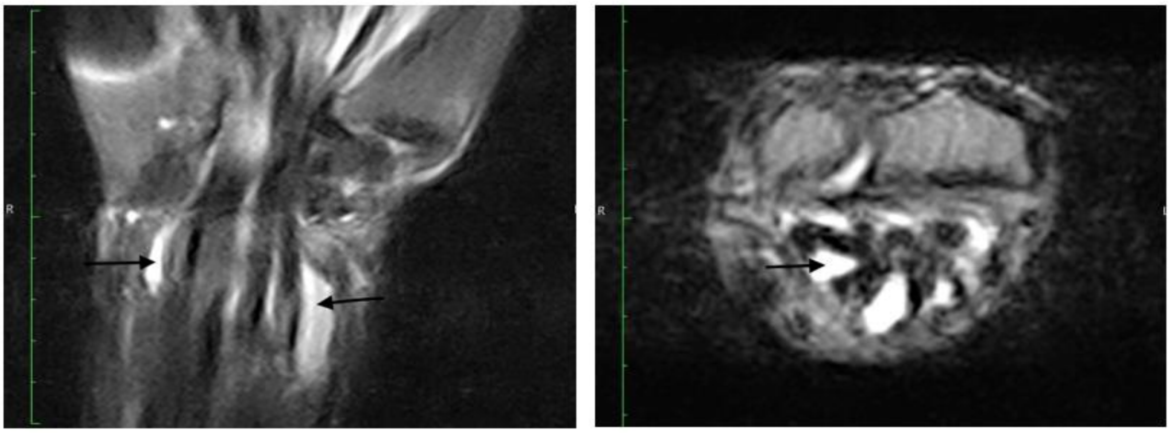

Supplementary Figure S2. Large volume of synovial fluid in the tendon sheaths of the flexor tendons seen when passing through the carpal tunnel in patient with SSc (**A**) coronal plane STIR (**B**) Axial plane STIR

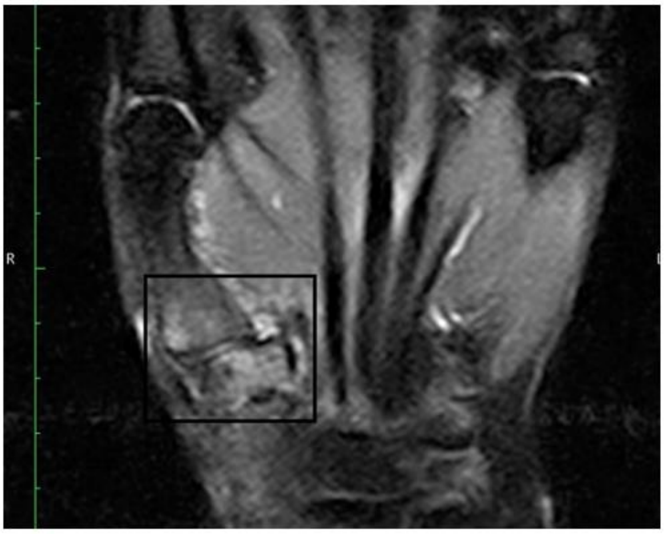

Supplementary Figure S3. CMC I subluxation with erosion on the articular facets, small amount of fluid inside the articular capsule and edema in the subchondral bone of both articular processes in patient with SSc

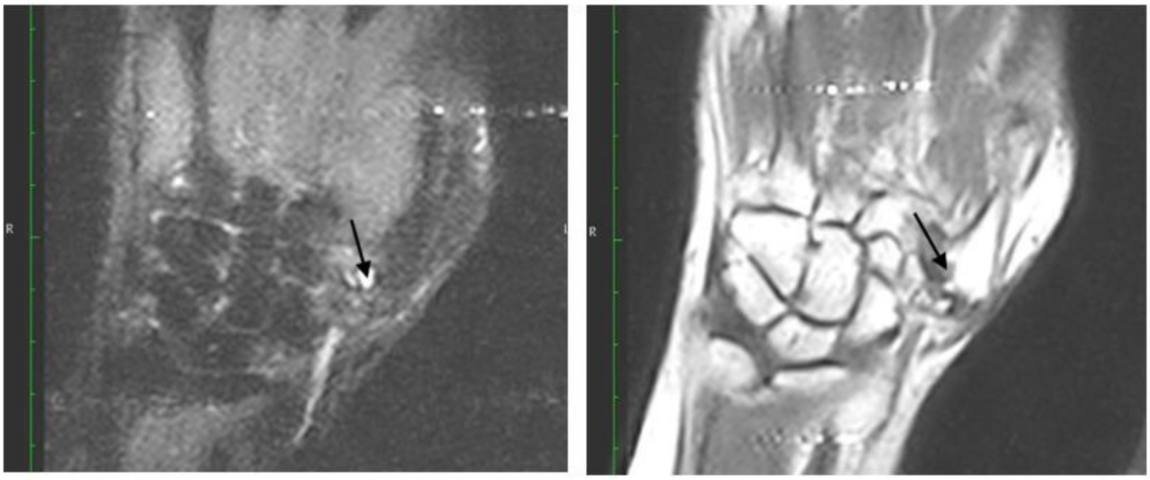

Supplementary Figure S4. Deep chondro-subchondral cyst on the articular facet of the head of the IV metacarpal bone in patient with SSc; STIR and T1 coronal plane

Supplementary Table S1. Average values of total MRI score of synovitis, edema and erosions in wrist and MCP joints of dominant hand in SSc and SSc subtypes.

**Localization (min. and max. values)**		**MRI score in SSc**	**MRI score in lcSSc**	**MRI score in dcSSc**	**P**
**Wrist**					
synovitis	(0-7)	2.69±2.29	2.46±2.16	3.23±2.52	0.222
erosions	(0-73)	6.58±10.89	5.56±6.71	9.00±17.17	0.371
bone edema	(0-33)	6.84±7.43	6.04±6.36	8.73±9.40	0.229
**MCP joints**					
synovitis	(0-12)	3.15±2.95	2.96±2.79	3.59±3.35	0.443
erosions	(0-80)	3.99±9.82	2.79±3.92	6.82±16.91	0.281
bone edema	(0-24)	4.04±4.76	3.87±4.56	4.45±5.28	0.651

Data are mean ± S.D. unless stated otherwise. SSc: systemic sclerosis; lcSSc: limited cutaneous systemic sclerosis; dcSSc: diffuse cutaneous systemic sclerosis; MCP: metacarpophalangeal.

Supplementary Table S2. Association of acro-osteolysis in hand MRI with erosion score and disease activity index (EScSG-AI) in patients with SSc

	**Acro-osteolysis on MRI**		
Parameter	**Yes**	**No**	P
EScSG-AI	5.23 ± 1.74 (5.75)	3.43 ± 1.77 (3.00)	0.0001
Erosion score (MR)	20.68 ± 34.17 (12.00)	6.85 ± 10.24 (2.00)	0.0106

Data are mean ± S.D. (medians) unless stated otherwise. EScSG-AI: Revised 2017 EUSTAR disease activity index.
